# Relationship between Rainfall, Fecal Pollution, Antimicrobial Resistance, and Microbial Diversity in an Urbanized Subtropical Bay

**DOI:** 10.1128/AEM.01229-20

**Published:** 2020-09-17

**Authors:** Nicole C. Powers, Hailey R. Wallgren, Sandra Marbach, Jeffrey W. Turner

**Affiliations:** a Texas A&M University–Corpus Christi, Corpus Christi, Texas, USA; Chinese Academy of Sciences

**Keywords:** 16S community, antimicrobial resistance, bacterial source tracking, enterococci, fecal pollution, rainfall

## Abstract

Urbanized bays are vulnerable to fecal bacterial pollution, and the extent of this pollution, in marine recreational waters, is commonly assessed by quantifying enterococcus concentrations. Recent reports have questioned the utility of enterococci as an indicator of fecal bacterial pollution in subtropical bays impaired by non-point source pollution, and enterococcus data alone cannot identify fecal bacterial sources (i.e., hosts). The purpose of this study was to assess relationships between rainfall, fecal bacterial pollution, antimicrobial resistance, and microbial diversity in an urbanized subtropical bay. Thus, a comprehensive bacterial source tracking (BST) study was conducted using a combination of traditional and modern BST methods. Findings show that rainfall was directly correlated with elevated enterococcus concentrations, including the increased prevalence of Enterococcus faecium, although it was not correlated with an increase in the prevalence of antimicrobial-resistant strains. Rainfall was also correlated with decreased microbial diversity. In contrast, neither rainfall nor enterococcus concentrations were directly correlated with the concentrations of three omnipresent host-associated fecal markers (i.e., human, canine, and gull). Notably, the human fecal marker (HF183) was inversely correlated with enterococcus concentrations, signifying that traditional enterococcus data alone are not an accurate proxy for human fecal waste in urbanized subtropical bays.

**IMPORTANCE** The presence of human enteric pathogens, stemming from fecal pollution, is a serious environmental and public health concern in recreational waters. Accurate assessments of fecal pollution are therefore needed to properly assess exposure risks and guide water quality policies and practices. In this study, the absence of a direct correlation between enterococci and source-specific human and animal markers disputes the utility of enterococci as an indicator of fecal pollution in urbanized subtropical bays. Moreover, the inverse correlation between enterococci and the human-specific marker HF183 indicates that recreational beach advisories, triggered by elevated enterococcus concentrations, are a misleading practice. This study clearly demonstrates that a multiparameter approach that includes the quantitation of host-specific markers, as well as analyses of microbial diversity, is a more effective means of assessing water quality in urbanized subtropical bays.

## Abstract (precis)

The presence of human enteric pathogens, stemming from fecal pollution, is a serious environmental and public health concern in recreational waters. Accurate assessments of fecal pollution are therefore needed to properly assess exposure risks and guide water quality policies and practices. In this study, the absence of a direct correlation between enterococci and source-specific human and animal markers disputes the utility of enterococci as an indicator of fecal pollution in urbanized subtropical bays. Moreover, the inverse correlation between enterococci and the human-specific marker HF183 indicates that recreational beach advisories, triggered by elevated enterococcus concentrations, are a misleading practice. This study clearly demonstrates that a multiparameter approach that includes the quantitation of host-specific markers, as well as analyses of microbial diversity, is a more effective means of assessing water quality in urbanized subtropical bays.

## INTRODUCTION

Fecal waste is a common pollutant in coastal marine environments (1). Previous studies have shown that fecal pollution can increase the prevalence of human enteric pathogens (e.g., Clostridium perfringens, enteroviruses, hepatitis A viruses, Norwalk viruses, and adenoviruses) (2, 3) and fundamentally alter the microbial community composition in aquatic environments (4, 5). The occurrence of fecal pollution poses a serious threat to human and environmental health, as the increased prevalence of these pathogens has long been correlated with an increased risk of illness in humans and marine life (2, 6).

Stormwater runoff associated with rainfall events is a significant source of fecal pollution (7). In urbanized bays, stormwater can aid in the land-to-sea transport of pollution stemming from leaks in aging sewage and septic infrastructure (8, 9). As a result of coastal development and the loss of vegetative landscape, even a small rainfall event can create a large pulse of stormwater (10), whereas extreme weather events such as tropical storms and hurricanes can cause catastrophic flooding and generate massive pulses of stormwater (11). Stormwater runoff can also carry excess nutrients, pesticides, residual antimicrobial compounds, petroleum-based pollutants, and heavy metals that may adversely affect coastal systems (12–14).

Antimicrobial resistance in aquatic environments has been linked to fecal waste, which is a source of antimicrobial-resistant bacteria and residual antimicrobial compounds (15, 16). Runoff from landfills and sludge applied to land can also transport antimicrobial-resistant bacteria and residual antimicrobial compounds to coastal systems (17). The presence of resistance genes and residual antimicrobial compounds has been shown to select for the evolution and survival of resistant bacteria (15). In turn, the selection and spread of resistance can disturb the structure and function of aquatic microbial communities (18), and recreational exposure to impacted aquatic environments can lead to harmful and difficult-to-treat bacterial infections (19).

Fecal pollution is routinely monitored through the measurement of fecal indicator bacteria (FIB) including total coliforms, fecal coliforms, Escherichia coli, C. perfringens, and enterococci (1, 20). In coastal marine environments, enterococci, a group of enteric Gram-positive bacteria, are commonly used as FIB (21). For example, in Texas (USA), the Texas Beach Watch Program routinely monitors only enterococci as an indicator of marine water quality (22). However, numerous studies have shown that enterococci are not an ideal indicator, as they are often detectable in pristine environments and can persist and multiply long after their initial introduction into the environment (23, 24). Studies have also shown that enterococci are not accurate predictors of human health risks in locations impaired by non-point sources of pollution (25, 26). Additionally, enterococci are not a host-specific indicator and thus cannot be used to accurately determine the source of fecal pollution (27).

The Source Identification Protocol Project (SIPP) was conducted to test the suitability of 41 indicators of fecal pollution (27–30). The authors concluded that the PCR-based quantitation of host-associated molecular markers is the most accurate and informative method for assessing and tracking fecal pollution in the environment (27–30). The human-associated *Bacteroides* marker HF183 (31, 32), the gull-associated *Catellicoccus* marker LeeSeaGull (33–36), and the canine-associated *Bacteroidales* marker DogBact (26, 37) were among the most specific and sensitive and are, therefore, an improvement over traditional FIB for detecting fecal contamination.

To assess the relationship between rainfall, fecal pollution, antimicrobial resistance, and microbial diversity, we conducted a comprehensive bacterial-source tracking (BST) study in an urbanized subtropical bay. The objectives of this study included (i) measuring enterococcus concentrations, (ii) assessing the antimicrobial resistance profiles among Enterococcus faecium isolates, (iii) quantifying host-associated markers of human, canine, and gull fecal pollution, and (iv) characterizing the microbial diversity. We hypothesized that rainfall would be correlated with increases in enterococcus concentrations, the prevalence of antimicrobial resistance, and the abundance of host-associated fecal pollution markers.

## RESULTS

### Sample collection and environmental parameters.

**FIG 1:**
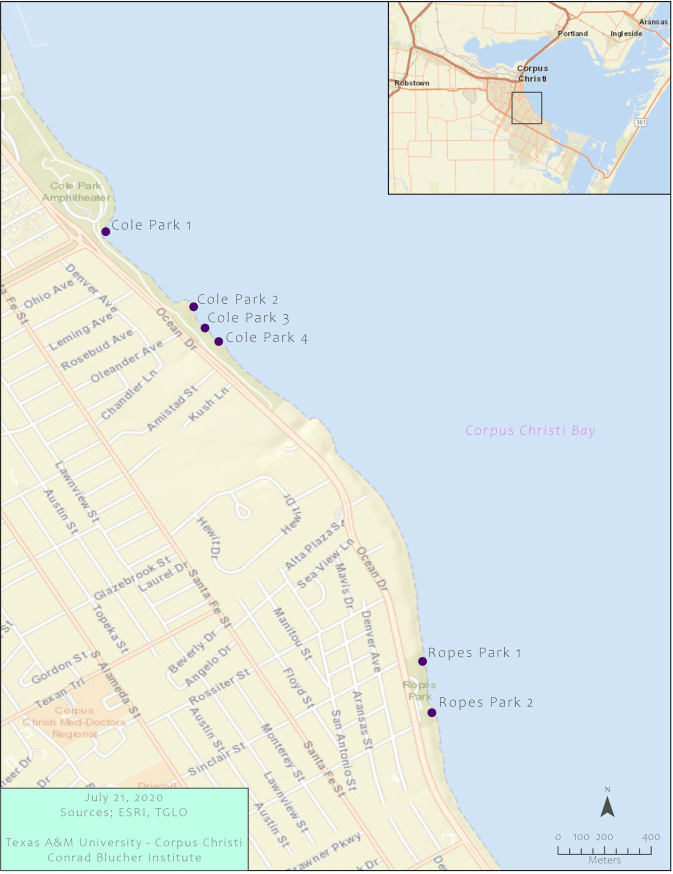
Map of the six sampling sites in Cole and Ropes Parks in Corpus Christi Bay, TX, USA. Ropes Park 1 and Cole Park 1, 3, and 4 are located within 5 m of storm drain outfalls. (Map by Jose Pilartes-Congo at the TAMU-CC Conrad Blucher Institute.)

Water sampling (120 total samples) occurred at four sites in Cole Park (TX259473) and two sites in Ropes Park (TX821303) (Fig. 1). Four sites were located within 5 m of stormwater outfalls. A total of 20 sampling events occurred, including six wet-loading events (6 events, 6 sites, 36 samples) and 14 dry-loading events (14 events, 6 sites, 84 samples). All wet-loading events were preceded by a minimum of 7 days without rainfall.

Throughout the study period, water temperature varied seasonally from 10.7 to 32.5°C, salinity ranged from 28.49 to 38.3 ppt, dissolved oxygen ranged from 4.04 to 11.31 mg ml^−1^, and pH ranged from 7.35 to 8.27. Water transparency varied from 0.063 to more than 1.21 m, specific conductance varied from 4,4094 to 5,7627 μS cm^−1^, and the number of days since precipitation occurred ranged from 0 to 33.

### Quantifying enterococci.

**FIG 2:**
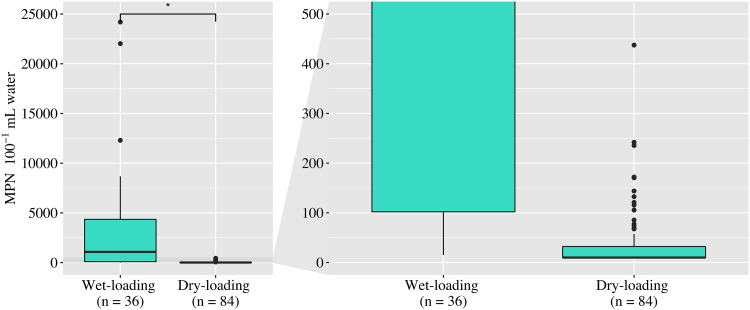
Concentrations of enterococci after wet-loading and dry-loading sampling events. The panel on the right shows an expanded view of the dry-loading samples. Significantly higher concentrations of enterococci were detected after wet-loading events (cendiff test; *, *P* < 0.01).

Enterococci were enumerated from a total of 120 water samples in duplicate via Enterolert testing. Concentrations ranged from a most probable number (MPN) of fewer than 10 to more than 24,196 100 ml^−1^ (lower and upper limits of detection, respectively) (Table 1). Samples collected after rainfall had significantly higher levels of enterococci (Fig. 2) (cendiff test, *P* < 0.01). Based on the cenken test, enterococcus concentrations were correlated with the following environmental parameters: the number of days since precipitation occurred (−0.440, *P* < 0.001), water transparency (−0.324, *P* < 0.001), pH (−0.145, *P* < 0.05), specific conductance (−0.143, *P* < 0.05), and salinity (−0.134, *P* < 0.05). Additionally, based on the cendiff test, higher enterococcus concentrations were correlated with water that was observed to be turbid rather than cloudy or clear (*P* < 0.001).

**TABLE 1:** Abundances of enterococci and the three host-associated molecular markers in wet- and dry-loading samples

Event type	Bacterial target	Abundance^*a*^			
		Minimum	Maximum	Mean	Median
Wet loading	Enterococci^*b*^	15.00	24,196.00	4,062.25	1,080.25
	Human marker^*c*^	66.67	3,294.43	385.13	190.00
	Canine marker	17.77	3,681.10	531.62	303.33
	Gull marker^*c*^	75.57	3,003.33	465.09	214.73

Dry loading	Enterococci^*b*^	<10.00	437.50	36.63	10.00
	Human marker^*c*^	95.53	3,680.00	771.32	358.33
	Canine marker	59.97	2,036.67	574.32	437.23
	Gull marker^*c*^	97.77	3,133.33	801.37	461.65

a Abundances are in MPN 100 ml^−1^ (enterococci) or gene copies 100 ml^−1^ (markers).

b Enterococci increased significantly after wet loading (*P* < 0.05).

c The marker increased significantly after dry loading (*P* < 0.05).

### Identifying *Enterococcus* species.

A total of 782 presumptive *Enterococcus* isolates were preserved, and 202 isolates were randomly selected for the duplex PCR assay to determine species; 109 isolates were from wet-loading events and 93 isolates were from dry-loading events. In total, 133 were identified as E. faecium, 32 were identified as E. faecalis, and the remaining 37 could not be identified with this assay. Multivariate analysis of variance (MANOVA) indicated that the majority of the identifiable *Enterococcus* isolates collected after rainfall were E. faecium (87 of 109; *P* < 0.01), while the occurrence of E. faecalis did not change significantly after rainfall. For this reason, the E. faecium isolates were chosen for antimicrobial susceptibility testing.

### Antimicrobial susceptibility.

All E. faecium isolates tested (*n* = 119; 80 from wet loading and 39 from dry loading) were susceptible to ampicillin and chloramphenicol, and only one wet-loading isolate was intermediately resistant to vancomycin. Eighteen of the wet-loading isolates and four of the dry-loading isolates were intermediately resistant to oxytetracycline, and one dry-loading isolate was fully resistant to this compound. There was no significant increase in the occurrence of antimicrobial resistance after wet loading in E. faecium isolates.

### Membrane filtration and DNA extraction.

Successful DNA isolation from all 120 water samples was confirmed with a BioSpectrometer (Eppendorf, Hamburg, Germany), which was used to determine the quality (*A*_260_/*A*_280_) and quantity (in nanograms per microliter) of DNA. However, due to funding limitations, not all samples were analyzed. DNA isolated from 54 samples, including one sampling event per month from May 2017 to January 2018 (three wet-loading events with 18 samples and six dry-loading events with 36 samples), was analyzed for the presence of host-associated markers, while DNA isolated from 72 samples (six wet-loading events with 36 samples and six dry-loading events with 36 samples) was analyzed by 16S rRNA gene sequencing.

### Bacterial source tracking.

**FIG 3:**
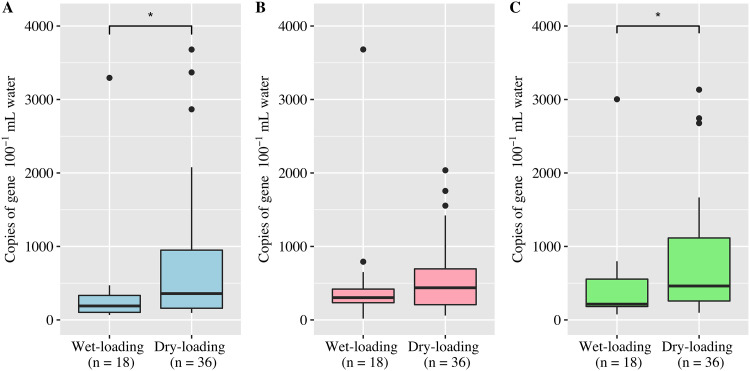
Concentrations of the host-associated markers from wet-loading and dry-loading samples. (A) Human marker (blue); (B) canine marker (pink); (C) gull marker (green). The human and gull markers were significantly higher after dry-loading than wet-loading events (Welch’s *t* test; *, *P* < 0.05).

The three host-associated markers tested in this study (human, canine, and gull) were detected in all of the water samples (*n* = 54). On average, the gull marker was the most abundant, followed by the human and canine markers (Table 1). The *t* tests showed that the human and gull markers were significantly (*P* < 0.05) higher after dry-loading events (Fig. 3). In contrast, the canine marker was not correlated with wet or dry loading (Fig. 3). All host-associated markers were positively correlated with each other, although only the human marker was negatively correlated with enterococci (Pearson’s correlation coefficient, −0.313; *P* < 0.05) (Table 2). The best-fit linear model for human marker included pH and water color (adjusted *R*^2^, 0.158; *P* < 0.05). The canine model included pH, water surface conditions, water odor, and current weather conditions (adjusted *R*^2^, 0.403; *P* < 0.01). The gull model included pH, water odor, and current weather conditions (adjusted *R*^2^, 0.292; *P* < 0.01). Despite the negative correlation between enterococci (MPN) and human markers, MPN was not a significant predictor in any of the models.

**TABLE 2:** Pearson’s correlation coefficients for enterococci (MPN 100 ml^−1^) and the abundance of host-associated molecular markers (gene copies 100 ml^−1^)

Organism or marker	Correlation coefficient^*a*^		
	Gull	Canine	Human
Enterococci	—	—	−0.313*
Human	0.608**	0.522**	
Canine	0.768**		

a *, *P* < 0.05; **, *P* < 0.01; —, nonsignificant correlation.

### Microbial community analysis.

**FIG 4:**
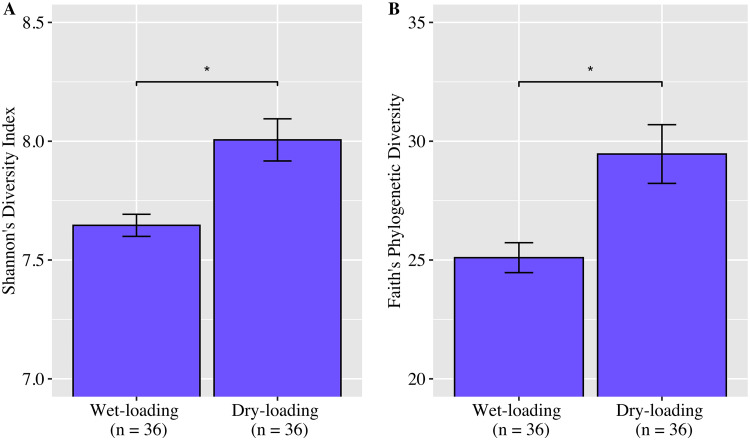
Alpha diversity metrics of wet-loading and dry-loading samples. (A) Shannon’s diversity index; (B) Faith’s phylogenetic diversity. Error bars represent the standard errors of the means. The dry-loading samples were significantly more diverse than the wet-loading samples (Kruskal-Wallis pairwise H test; *, *P* < 0.01).

**FIG 5:**
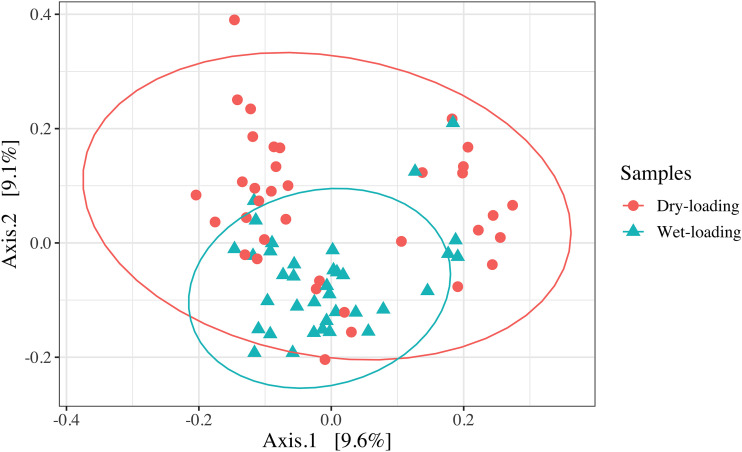
Principal-coordinate analysis (PCoA) based on unweighted UniFrac distance values showing beta diversity of dry-loading (red circles) and wet-loading (blue triangles) samples. The ellipses represent 95% confidence intervals for each sampling type. The community compositions of wet-loading and dry-loading samples were significantly different (pairwise PERMANOVA; *P* < 0.01).

**FIG 6:**
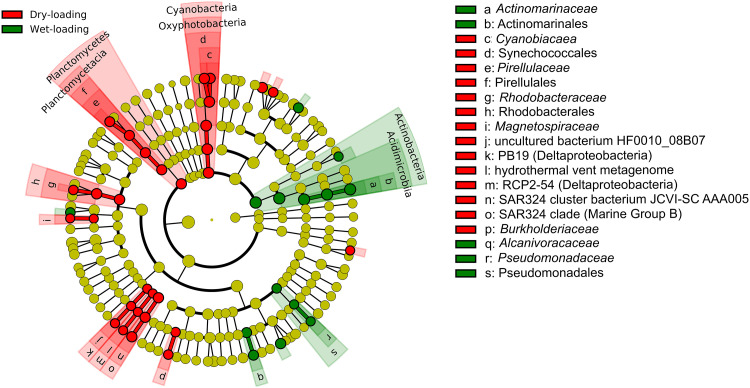
Linear discriminant analysis (LDA) effect size (LEfSe) cladogram depicting the taxa enriched in the dry-loading (red) and wet-loading (green) samples (*P* < 0.01).

Kruskal-Wallis pairwise H tests comparing Shannon’s diversity index and Faith’s phylogenetic diversity (FPD) between the samples collected after wet and dry loading showed that alpha diversity was significantly lower in the wet-loading samples (*P* < 0.01) (Fig. 4). Similarly, principal-coordinate analysis (PCoA) of unweighted UniFrac distance values (accounting for 18.7% of variance) and subsequent pairwise permutational multivariate analysis of variance (PERMANOVA) confirmed that wet- and dry-loading communities were significantly different (*P* < 0.01) (Fig. 5). The linear discriminant analysis (LDA) effect size (LEfSe) further investigated how the wet- and dry-loading communities differed. Results showed that several taxa (e.g., *Cyanobacteria*, *Planctomycetes*, *Rhodobacterales*, Deltaproteobacteria, and *Burkholderiaceae*) were enriched in the dry-loading communities (*P* < 0.01) (Fig. 6). In contrast, fewer taxa (e.g., *Actinobacteria*, *Pseudomonadales*, and *Alcanivoracaceae*) were enriched in the wet-loading communities.

## DISCUSSION

Fecal waste is a widespread pollutant in urbanized bays (9, 38, 39). The extent of fecal pollution in marine surface waters is commonly assessed by measuring FIB, including total coliforms, fecal coliforms, Escherichia coli, C. perfringens, and enterococci (1, 20). However, the detection and quantitation of FIB cannot identify the sources of fecal pollution (20). Furthermore, traditional FIB indicators cannot explain the larger consequences of fecal pollution, such as increases in antimicrobial resistance or decreases in microbial diversity. To address these limitations, this study used a combination of traditional and modern BST methods to assess relationships between rainfall, fecal pollution, antimicrobial resistance, and microbial diversity in an urbanized subtropical bay.

Enterococci are routinely monitored throughout coastal Texas as a proxy for water quality by the Texas Beach Watch Program; therefore, this study assessed enterococci in lieu of other commonly used FIB. Enterococcus concentrations frequently exceeded the recreational water quality criterion established by the U.S. Environmental Protection Agency (EPA) (single-sample standard of 104 CFU 100 ml^−1^) by 40-fold, and the highest concentrations were measured after rainfall events. In agreement, elevated enterococcus concentrations were also inversely correlated with water transparency, pH, and salinity. Conversely, in the absence of rainfall, enterococcus concentrations were consistently lower than the EPA criterion. Rainfall and stormwater runoff have been shown to increase coastal bacteria concentrations and also decrease water transparency, pH, and salinity (40). The sources of bacteria, however, were unknown, as enterococci are not host specific (20), have been detected in uncontaminated locations (24), and have been shown to persist for long periods without a host (23).

To determine potential sources of fecal pollution, an increasing number of BST studies have included the detection of host-associated molecular markers (27, 41, 42). In this study, human-, canine-, and gull-associated markers were ubiquitous and occasionally abundant, but none were positively correlated with enterococcus concentrations; in contrast, the human-associated marker HF183 was inversely correlated with enterococci. This lack of direct correlation indicates that enterococci were not an accurate proxy for human-, canine-, or gull-associated fecal pollution in this subtropical bay. Previous studies have reported a direct correlation between enterococci and human-associated *Bacteroides* markers (43), an inverse correlation (44), and no correlation (9). Previous studies have also questioned the use of enterococci as the primary FIB due to the lack of a dose-response relationship between gastroenteritis and enterococcus concentrations among recreational bathers in subtropical waters without known point sources of sewage (25, 26).

Rainfall and its accompanying stormwater runoff have been identified as important drivers of fecal pollution and waterborne illness (7, 8, 45). However, in this study, rainfall and the human-associated marker were inversely related. This finding suggests that stormwater may not be the major source of human fecal waste in this system; rather, the abundance of the human-associated marker during dry loading may indicate that human fecal waste was omnipresent but diluted during storm events. Abundant fecal waste during dry loading could result from an aging sanitary sewage infrastructure, as seepage from leaks and breaks would be a continuous source of pollution (8, 9). Alternatively, the high concentrations of fecal pollution after dry loading could also be attributed to reduced dilution that would normally accompany freshwater inputs during wet-loading events (46, 47). These results suggest that recreational bathers are at higher risk of exposure to human fecal waste during dry weather conditions. Thus, rainfall-related beach advisories triggered by increased enterococcus concentrations may not protect recreational bathers from human fecal pollution.

Canines and gulls have been determined to be major sources of fecal pollution in marine surface waters (48). As the presence of animal-associated fecal contamination can indicate the presence of zoonotic pathogens, including these fecal markers in comprehensive BST studies is essential. Rainfall was not correlated with gull fecal pollution in this study; rather, the gull marker was present constantly and was, on average, the most abundant of the markers. While the concentration of the gull marker diminished after rainfall, the canine marker concentration remained relatively constant. The steady canine pollution could have stemmed from domestic and feral canine waste in coastal neighborhoods and parks that washed into stormwater drains after rainfall. Canine fecal pollution can be mitigated with proper education regarding pet waste disposal (49), and gull abatement programs focused on preventing the loitering of gulls have resulted in significant decreases in gull fecal pollution (50).

In addition to fecal waste, rainfall-associated runoff can transport antimicrobial-resistant microbes and residual antimicrobials to surrounding environments (51), and studies have shown that runoff can increase the occurrence of antimicrobial resistance (52, 53). In this study, rainfall was correlated with an increase in the occurrence of E. faecium, but we did not observe a significant increase in antimicrobial resistance in this species. The lack of resistance suggests that the enterococci did not stem from a human source and were instead environmental. A previous study detected higher levels of antimicrobial resistance genes in the environment after rainfall; however, this coincided with higher concentrations of both enterococci and the HF183 human-associated fecal marker (51). A shotgun metagenomic approach could have revealed rainfall-correlated differences in the abundance of antimicrobial resistance genes, but that analysis was outside the scope of this study.

Pulses of stormwater have been shown to alter the composition of bacterial (54) and viral (55) communities. Here, through a combination of four community-based metagenomic analyses (i.e., Shannon’s diversity, FPD, PCoA, and LEfSe), we have shown that (i) wet- and dry-loading communities were significantly different, (ii) wet-loading communities were less diverse, and (iii) specific genera were enriched (i.e., differentially more abundant) in each community. Higher microbial diversity is thought to promote community stability and functional resilience after disturbance (56). More frequent or more extreme rainfall events could, therefore, impair the stability and resilience of this system. This finding is of particular importance, seeing that the Fourth National Climate Assessment predicted that the northern Gulf of Mexico (nGOM) will experience increased extreme rainfall events, including tropical depressions, tropical storms, and hurricanes (57). Urban systems are especially vulnerable to extreme rainfall, which can overwhelm and damage urban infrastructure. Ultimately, per the recommendations of the climate assessment, forward-looking infrastructure designs and practices will be necessary to safeguard urban systems from ongoing and future climate risks.

In conclusion, this study demonstrated that rainfall was correlated with increased enterococcus concentrations and decreased microbial diversity. Rainfall was not, however, correlated with the detection of human, canine, or gull fecal pollution. Likewise, rainfall was not correlated with an increase in E. faecium antimicrobial resistance. The inverse relationship between enterococci and the HF183 human marker suggests that the presence of elevated enterococci is not an accurate indication of human fecal contamination in this system. Moreover, in agreement with similar BST studies (42, 58), these findings suggest that the utility of fecal pollution indicators may be location specific, and thus, independent analyses should be conducted in areas of concern to identify suitable indicators.

## MATERIALS AND METHODS

### Sample collection and environmental parameters.

The city of Corpus Christi (total area, 504 mi^2^; population, 326,554) is located on Corpus Christi Bay along the Texas segment of the nGOM, United States (59, 60). Elevated enterococcus concentrations have been reported at recreational parks in Corpus Christi Bay (61–63), with concentrations frequently exceeding the EPA’s single-sample standard of 104 CFU of enterococci per 100 ml of water (64). Thus, six stations in Corpus Christi Bay, including four stations in Cole Park (TX259473) and two stations in Ropes Park (TX821303), were selected as sampling sites for this study (Fig. 1). Two-liter surface water samples were collected biweekly from May 2017 through January 2018, and additional water samples were collected after major rainfall events. Water samples were collected in autoclave-sterilized polypropylene bottles, stored on ice, and processed within 4 h of collection. Sampling events that occurred within 24 h of rainfall were considered wet-loading events, and the remaining events were considered dry-loading events.

Physical parameters (water temperature [degrees Celsius], salinity [parts per thousand], dissolved oxygen [milligrams per milliliter], pH, and specific conductance [microsiemens per centimeter) were measured with a YSI 556 Multi Probe system (YSI Incorporated, Yellow Springs, OH, USA). A Kestrel wind meter (Kestrel Instruments, Boothwyn, PA, USA) was used to measure wind speed (miles per hour) and air temperature (degrees Celsius), and a transparency tube (Ben Meadows, Janesville, WI, USA) was used to measure water transparency (meters). Precipitation data (inches per day and the number of days since precipitation) were retrieved from the nearest weather station (KTXCORPU268). Additional environmental parameters (e.g., water color, odor, surface conditions, and current weather conditions) were observed and recorded as categorical variables. Specifically, the color of the water was classified as colorless, green, tan, or brown, and the odor was classified as odorless, fishy, rotten egg, or sewage. Water surface conditions included calm water, ripples, waves, and white caps, whereas overall water conditions included clear water and debris-, foam-, or scum-laden water. Current weather conditions included rain, overcast, cloudy, or clear sky. Finally, the overall water clarity was classified as clear, cloudy, or turbid. These categorical variables were added in consideration of citizen scientist studies where these and similar variables were found to be significant predictors of water quality (65–67).

### Quantifying enterococci.

Enterococci were quantified using the Enterolert test (IDEXX Laboratories, Westbrook, ME, USA) at the Corpus Christi Nueces County Public Health District Laboratory (CCNCPHDL), which is accredited by the National Environmental Laboratory Accreditation Program (NELAP). Duplicate 100-ml aliquots of each water sample were provided to CCNCPHDL in accordance with the Texas Beach Watch Program testing criteria for marine water (22). Enterococcus concentrations were reported as the most probable number (MPN) of enterococci per 100 ml. Due to the lower limit of detection (MPN of <10) in the Enterolert test, statistical correlation tests for censored data were computed on R (version 3.3.1) and RStudio (version 0.99.903) with the use of the NADA package (68). Specifically, the cenken test was used to calculate Kendall's tau correlation coefficient of enterococci with continuous environmental variables, and the cendiff test was used to test the association of enterococci with categorical variables. Additionally, enterococcus concentrations from wet-loading samples were compared to the concentrations from dry-loading samples using the cendiff test, which served as a censored *t* test.

### Identifying *Enterococcus* species.

Presumptive *Enterococcus* colonies were isolated via the EPA 1600 membrane filtration method (69). Duplicate 100-ml water samples were filtered aseptically through 0.45-μm mixed cellulose ester (MCE) membrane filters (47 mm in diameter; Millipore Sigma, Bedford, MA, USA), placed on sterile membrane-*Enterococcus* indoxyl-β-d-glucoside (mEI) agar plates (Beckton, Dickinson and Company, Sparks, MD, USA), and incubated at 41°C for 24 h. Up to four CFU with blue halos were randomly selected and streaked for isolation on brain heart infusion agar (BHIA) plates (Beckton, Dickinson and Company, Sparks, MD, USA) at 37°C for 24 h. Isolated colonies (*n* = 782) were transferred to brain heart infusion broth (BHIB) (Beckton, Dickinson and Company, Sparks, MD, USA) and grown at 37°C with shaking (120 rpm) for 24 h before being cryopreserved at −80°C in a 25% (final concentration) glycerol solution. DNA was isolated via a 5-min boil lysis method (70). PCR primers targeting the species-associated alleles of the *sodA* gene (Table 3) were used to identify E. faecalis and E. faecium isolates (71) (*n* = 202/782 randomly selected isolates) with the following cycling conditions (Table 4): 5-min hold at 95°C, followed by 40 cycles of 96°C for 5 s, 45°C for 5 s, and 68°C for 10 s, followed by a 1-min hold at 72°C. MANOVA was used to determine if the presence of either species was correlated with wet or dry loading.

**TABLE 3:** Primer sequences and gene targets for PCR assays utilized in this experiment

Target	Primer sequence	Reference(s)
Enterococcus faecalis (*sodA* gene)	Forward: 5′-ACTTATGTGACTAACTTAACC-3′	71
	Reverse: 5′-TAATGGTGAATCTTGGTTTGG-3′	
Enterococcus faecium (*sodA* gene)	Forward: 5′-GAAAAAAACAATAGAAGAATTAT-3′	71
	Reverse: 5′-TGCTTTTTTGAATTCTTCTTTA-3′	
Human-associated *Bacteroides* HF183^*a*^	Forward primer: 5′-ATCATGAGTTCACATGTCCG-3′	31, 32
	Reverse primer: 5′-TACCCCGCCTACTATCTAATG-3′	
Canine-associated *Bacteroidales* DogBact^*b*^	Forward primer: 5′-CGCTTGTATGTACCGGTACG-3′	26, 37
	Reverse primer: 5′-CAATCGGAGTTCTTCGTG-3′	
Gull-associated *Catellicoccus* LeeSeaGull^*c*^	Forward primer: 5′-AGGTGCTAATACCGCATAATACAGAG-3′	33–36
	Reverse primer: 5′-GCCGTTACCTCACCGTCTA-3′	
16S rRNA	Forward: 515fMod, 5′-GTGYCAGCMGCCGCGGTAA-3′	81
	Reverse: 806rMod, 5′-GGACTACNVGGGTWTCTAAT-3′	

*^a^*Accession number AY618281.1.

*^b^*Accession number AY695700.1.

*^c^*Accession number NR_042357.

**TABLE 4:** Cycling conditions for PCR assay of the *sodA* gene in *Enterococcus*

Step	Temp (°C)	Time^*a*^	Ramp rate	No. of cycles
Enzyme activation	95	5:00	2°C s^−1^	1
Denaturation	96	0:05	2°C s^−1^	40
Annealing	45	0:05	2°C s^−1^	40
Extension	68	0:10	2°C s^−1^	40
Final extension	72	1:00	2°C s^−1^	1

a In minutes:seconds.

### Antimicrobial susceptibility.

Due to funding and resource limitations, 119 of the 133 E. faecium isolates were tested in triplicate for antimicrobial susceptibility by disk diffusion in accordance with the Clinical and Laboratory Standards Institute (CLSI) protocol (72). Four antimicrobial compounds belonging to different classes were tested: ampicillin (10 μg), vancomycin (30 μg), chloramphenicol (30 μg), and oxytetracycline (30 μg) (Becton, Dickinson, Franklin Lakes, NJ, USA). The concentration of each compound was chosen based on CLSI recommendations (72). Isolates were grown on BHIA at 37°C overnight and diluted with a 0.45% sterile saline solution to approximate a 0.5 McFarland standard. Sterile cotton swabs (Puritan, Guilford, ME, USA) were used to create bacterial lawns on Mueller-Hinton agar plates (Beckton, Dickinson and Company, Sparks, MD, USA). The plates were divided into equal quadrants and the antimicrobial-infused disks were aseptically placed in the center of each quadrant. A blank disk containing only the saline solution vector was included as a negative control. The plates were incubated at 37°C (16 to 20 h for ampicillin, chloramphenicol, and oxytetracycline; 24 h for vancomycin). The zones of inhibition were measured in triplicate for each disk, and results were recorded and classified as susceptible, intermediate, or resistant. E. faecalis ATCC 29212 (susceptible to ampicillin, vancomycin, and high levels of gentamicin and streptomycin) was used as the control strain (73). The results were analyzed with a Wilcoxon-Mann-Whitney U test for ordinal categorical data to determine if antimicrobial resistance was correlated with wet or dry loading.

### Membrane filtration and DNA extraction.

DNA was extracted from water samples (*n* = 120) for BST and community structure analyses. Duplicate 100-ml water samples were filtered through 0.45-μm MCE filters (47-mm diameter) (Millipore Sigma, Bedford, MA, USA). Previous studies have shown that none of the standard pore sizes (i.e., 0.45, 0.22, and 0.1 μm) are capable of capturing the entire bacterial community (74). This pore size allowed the volume filtered (100 ml) to be standardized across all analyses (i.e., enterococci, bacterial source tracking, and microbial community analysis). This pore size also allowed the capture of enterococci as well as *Bacteroides* and *Catellicoccus* species (69, 75–77). The filters were placed in sterile 5-ml centrifuge tubes, Parafilm was applied, and filters were stored at −80°C for no longer than 14 days. Filters were aseptically cut into small strips, and DNA was extracted with a DNeasy PowerSoil kit (Qiagen, Valencia, CA, USA) following manufacturer’s instructions. The DNA was assessed for quality (*A*_260_/*A*_280_) and quantity (nanograms per microliter) using a BioSpectrometer (Eppendorf, Hamburg, Germany) and stored at −20°C.

### Bacterial source tracking.

To determine the most probable sources of fecal pollution, the DNA extracts were tested for the presence of molecular markers of bacterial strains associated with the feces of humans, canines, and gulls (Table 3). The quantity of each marker was assessed individually with a droplet digital PCR (ddPCR) assay following a previously established protocol (78). Briefly, a total of 54 water samples, collected from May to January (one sampling event per month), including three wet-loading and six dry-loading events, were tested in triplicate for the presence of the host-associated molecular markers. Each ddPCR mixture consisted of the following components: 10 μl EvaGreen Supermix (1× final concentration), 1 μl forward primer (0.25 μM), 1 μl reverse primer (0.25 μM), 3 μl of DNA, and 5 μl PCR-grade, nuclease-free water. Each ddPCR run included positive controls for each marker in the form of synthetic gBlock gene fragments (Integrated DNA Technologies, Skokie, IL, USA) (accession numbers are shown in Table 3) and no-template controls (NTCs) that contained sterile, nuclease-free water in place of DNA. Droplets were generated with a QX200 droplet generator (Bio-Rad Laboratories, Hercules, CA, USA), and markers were amplified with a Bio-Rad C1000 Touch thermal cycler (Bio-Rad Laboratories, Hercules, CA, USA) with the following conditions (Table 5): 5-min hold at 95°C, followed by 40 cycles of 95°C for 30 s and 59°C for 1 min, followed by a 5-min hold at 4°C and a final 5-min hold at 90°C. Following amplification, droplets were transferred to a QX200 droplet reader (Bio-Rad Laboratories, Hercules, CA, USA), and the markers were quantified with QuantaSoft software following the manufacturer’s instructions. The peaks from the NTCs were used to manually set the thresholds for positive droplets, and wells with fewer than 10,000 reported droplets were removed from the analysis. QuantaSoft reported the number of gene copies per microliter of each ddPCR, which was converted to gene copies per 100 ml of water with equation 1:

(1) $$X_{\text{total}}\text{ = }\frac{\text{(}X_{n}\text{)(20 μl)}}{\text{(3 μl)(50 μl)}}$$

The final concentration of each host-associated marker (gene copies 100 ml^−1^) is indicated by *X*_total_, and *X_n_* is the marker concentration of the PCR reaction (gene copies per microliter); 20 μl is the total PCR volume, 3 μl is the volume of DNA in each PCR mixture, and 50 μl is the total DNA volume from each extraction.

**TABLE 5:** Cycling conditions for ddPCR assay of the host-associated molecular markers

Step	Temp (°C)	Time^*a*^	Ramp rate	No. of cycles
Enzyme activation	95	5:00	2°C s^−1^	1
Denaturation	95	0:30	2°C s^−1^	40
Annealing/extension	59	1:00	2°C s^−1^	40
Signal stabilization	4	5:00	2°C s^−1^	1
	90	5:00	2°C s^−1^	1

a In minutes:seconds.

The concentrations of host-associated markers (gene copies per 100 ml water) and enterococci (MPN per 100 ml water) were log transformed, and the normality of the transformed data was confirmed through visualization of quantile-quantile plots using the qqnorm function in RStudio. Correlations between the concentrations were tested by calculating Pearson’s correlation coefficient, and Welch’s *t* test determined if marker concentrations varied significantly between wet- and dry-loading events. Multiple linear regressions, computed using the lm function in RStudio, were used to test if environmental parameters were correlated with marker concentrations. For this purpose, a full model was generated for each of the host-associated markers to include all of the environmental variables measured during sampling. The full models were then assessed for collinearity with the VIF function from the car package (79) and variables with a GVIF^[1/(2·DF)]^ of >2.0 were removed. Model averaging was then performed using the dredge function from the MuMIn package (80). The exhaustive lists of potential models were ranked based on Akaike information criterion corrected (AICc) values, and models with an AICc 2.0 greater than the top model were eliminated. If multiple models remained, they were subsequently ranked based on adjusted *R*^2^ values. The final models were tested for normality using the qqplot function.

### Microbial community analysis.

The remaining DNA extracts were used to characterize the microbial communities of 36 wet-loading and 36 dry-loading samples. For this purpose, the V4 region of the 16S rRNA gene was amplified (81) (primers shown in Table 3) with a HotStarTaq Plus master mix kit (Qiagen, Valencia, CA, USA) with the following cycling conditions (Table 6): 3-min hold at 94°C, followed by 30 cycles of 94°C for 30 s, 53°C for 40 s, and 72°C for 1 min, followed by a 5-min hold at 72°C. Successful amplification was confirmed through visualization of PCR products in 2% agarose gels. The samples were then pooled and purified with Ampure XP beads (Beckman Coulter, Indianapolis, IN, USA) to create the sequencing library. DNA sequencing was performed on an Illumina HiSeq platform with 250-bp paired-end (PE) chemistry at Molecular Research LP (Shallowater, TX, USA). Bar codes were removed from the raw sequence reads with QIIME (version 1.9), and QIIME2 (version 2018.11) was used for subsequent steps of the analysis (82, 83). Briefly, the DADA2 plugin (84) was used to demultiplex, denoise, and dereplicate the reads, trim them to a length of 241 bp, and remove chimeric sequences. Next, the sequences were aligned with MAFFT (85) and filtered with default settings. The SILVA 132 release database (86) was imported and trained based on the target sequences of the 515 forward and 806 reverse modified primers (81) using a naive Bayes classifier in QIIME2 (fit-classifier-naïve-bayes command). Taxonomy was assigned based on the database, and features mapped to chloroplast or mitochondrial DNA were removed with the taxon filter-table command. A phylogenetic tree was inferred using FastTree (87) and rooted with default QIIME2 settings for use in the downstream diversity analyses. Alpha diversity (Shannon’s diversity index and Faith’s phylogenetic diversity [FPD]) and beta diversity (unweighted UniFrac distance values) were calculated using the q2-diversity plugin. The Kruskal-Wallis pairwise H test was used to test for correlation between the wet-loading and dry-loading alpha diversity values. Unweighted UniFrac distance values were used to generate a PCoA to visualize differences in beta diversity, using Phyloseq (version 1.30.0) (88). Community structure differences between wet- and dry-loading samples were analyzed using PERMANOVA. The differential abundance of microorganisms detected in wet- versus dry-loading communities was determined via linear discriminant analysis (LDA) effect size (LEfSe) (89). This test was computed using the LEfSe tool on the Galaxy server (https://huttenhower.sph.harvard.edu/galaxy/). Genera that made up more than 0.1% of the communities’ relative abundance were analyzed using default settings with the significance threshold set to a *P* value of <0.01.

**TABLE 6:** Cycling conditions for PCR assay of the V4 region of the 16S rRNA gene for community analysis

Step	Temp (°C)	Time^*a*^	Ramp rate	No. of cycles
Enzyme activation	94	3:00	3.35°C s^−1^	1
Denaturation	94	0:30	3.35°C s^−1^	30
Annealing	53	0:40	3.35°C s^−1^	30
Extension	72	1:00	3.35°C s^−1^	30
Final extension	72	5:00	3.35°C s^−1^	1

a In minutes:seconds.
